# Material inventory dataset for residential buildings in Finland

**DOI:** 10.1016/j.dib.2023.109502

**Published:** 2023-08-18

**Authors:** Tapio Kaasalainen, Mario Kolkwitz, Bahareh Nasiri, Satu Huuhka, Mark Hughes

**Affiliations:** a Tampere University, School of Architecture, P.O.Box 600, FI-33014 Tampereen yliopisto, Finland; b Aalto University, Department of Bioproducts and Biosystems, Vuorimiehentie 1, FI-02150 Espoo, Finland

**Keywords:** Anthropogenic resources, Building stock, Construction materials, Domestic buildings, Housing stock, Material composition, Material contents, Material intensity coefficients

## Abstract

This dataset contains the material volumes, masses, and intensities for a total of 45 residential building cohorts in Finland from the 1940s to the 2010s. The specific building types included are one dwelling houses and blocks of flats. The data were drawn from representative case buildings and their derivatives. The data are primarily based on construction drawings, complemented by other documents such as bills of materials. The source material was mainly obtained from the archives of the building inspection authority of the city of Vantaa, Finland. Material volumes were derived from the construction drawings either directly from annotations or, when needed, by further measurements made based on the same material. For minor lacks of information in the original documents, documents of similar buildings and literature were consulted. A total of 26 buildings were inventoried directly. For each included combination of building type, construction decade, and bearing material these were the ones with the most common façade material. In addition, 19 buildings with the second most common façade material were formed based on these to represent the 45 cohorts. Material masses, and by extension intensities, were calculated based on the recorded volumes and typical densities of construction materials used in Finland. The material volumes, masses, and intensities per material and in total are presented as three spreadsheet tables, along with a description sheet, on three corresponding hierarchical levels of aggregation: per representative building, per vertical building level (foundations, basement, first storey, etc.), and per building part (floor, exterior walls, interior walls, etc.). Furthermore, they are distinguished between the building structure and complementary building components (windows and doors). The data can be used in academic, policy related, and practical investigations of the building stock, such as in evaluating the material consumption consequences of different spatial planning strategies on various levels or estimating the materials embedded in the built environment and their potential for capitalisation in the circular economy.

Specifications Table

**Table:** 

Subject	Architecture; Civil and structural engineering; Environmental engineering
Specific subject area	Anthropogenic material stocks and flows; Building stock research; Industrial ecology; Urban metabolism; Urban mining
Type of data	Table
How the data were acquired	Data were acquired by analysing representative residential buildings’ construction drawings (e.g. floor plans, section drawings, structural detail drawings) and related building permit documents submitted to the building inspection authority. The drawings and other documents were downloaded in PDF format from the building inspection authority's digital archives. The documents were inspected and cross-referenced with one another to determine the volume of each inventoried building material. When needed to account for lacking dimension annotations, scale drawings were imported into an architectural drawing software (Graphisoft ArchiCAD and Autodesk Revit) and measured.
Data format	Processed
Description of data collection	Data was collected for typical housing cohorts defined by construction decade, building type, main bearing material, and main façade material. For each building material, its volume and location in the building were recorded. Due to missing information, certain components such as fasteners and building services equipment were excluded. Based on the recorded volumes complemented by density and floor area information, material masses and intensities were included.
Data source location	City: Vantaa Country: Finland Vantaa latitude 60.29, longitude 25.04 Lupapiste Kauppa, https://kauppa.lupapiste.fi/ National Archives of Finland, https://astia.narc.fi/
Data accessibility	Repository name: Zenodo Data identification number: 10.5281/zenodo.8219915 Direct URL to data: https://zenodo.org/record/8219915

## 1 Value of the Data

• Material inventories of building stocks can help to quantify, understand, and manage the consequences of different spatial planning strategies (such as building replacement or infill development) on construction material consumption, demolition waste generation, and associated environmental impacts on different spatial levels, from neighbourhoods and cities to regions and states. In addition to researchers, they can also be used by e.g. city planners and municipal to national policy makers.
• These inventories can also help to estimate sizes of urban mines, i.e. materials stocked in existing buildings, which can act as secondary sources of building materials in the circular economy of the future. This kind of information can be of interest for public policymakers and companies in the field of circular construction, such as demolition / deconstruction companies and construction product (re)manufacturers.
• The current dataset, focusing on residential buildings – namely houses and blocks of flats – is the first of its kind published on the building stock in Finland, located in the Boreal (subarctic) zone and traditionally characterised by the use of wooden structures in low-rise housing construction. The data on houses and blocks of flats can be adapted for use as proxies for other Finnish residential building types, e.g. attached and row houses. The contents and format of the dataset can inform further inventorying efforts.
• The materials are recorded at a high level of detail with regard to not only their types but also their location in the building. Thus when applying the data to the larger building stock, in addition to the minimum of floor area, building type, and age, building geometry can be taken into account if the respective information is available. For example, building footprint and storey count in statistics or GIS data can be used to scale material amounts on different vertical levels. With height information, material amounts in walls can be further adjusted.
• Researchers in the fields of industrial ecology / material stocks and flows / urban metabolism as well as spatial planning and circular construction practitioners, e.g. architects, can reuse this data to avoid repetitive, labour-intensive bottom-up data collection. The granularity of the data enables also narrower focuses, from per building assessments to e.g. specific parts of buildings. Further data collection efforts on the Finnish building stock can be directed at non-residential buildings to complement this dataset.
• In addition to its main purpose, the dataset also contributes systematic understanding on Finnish residential buildings regarding their construction methods and materials. In doing so, the data can benefit building stock research, which aims at understanding characteristics of building stocks, and its applications, such as the study of repair, refurbishment, and renovation of building structures.

## 2 Objective

Material inventories of building stocks are necessary input data for bottom-up material stock and flow analyses (MSFA) of the built environment. Such data have previously been published for e.g. Canada [1], Germany [2], Japan [3], and Sweden [4], among other locations [5]. The current dataset was generated to facilitate the estimation of materials embedded in the Finnish residential building stock as well as flowing in and out of it through new construction and demolition. Due to the lack of information on the material contents of the Finnish building stock [6,7], previous MSFA studies on the built environment in Finland have had to (1) characterise stocks and flows of buildings by their number or floor area (e.g. Huuhka & Kolkwitz [8]; Kolkwitz et al. [9]), or (2) base their quantification of materials on datasets derived from other countries (e.g. Nasiri et al. [6]). In the absence of endogenous material indicators for the Finnish stock, the quantification of construction materials stocked in these buildings has not been as explicit or as reliable as would be ideal. The current dataset has been created to start bridging this gap in data.

## 3 Data Description

### 3.1 Scope and Granularity of the Data

The presented dataset [10] comprises an OpenDocument Spreadsheet (ODS) file *material_inventory_dataset_[version].ods* with four sheets, *data_building, data_building_level, data_building_part*, and *description*. The *data* sheets contain the volume, mass, and material intensity (kg/m^2^) of each recorded material (see Table 1), in each inventoried building (Table 2), and further distinguished by its location in the building (Table 3).

**Table 1:** Materials included in the dataset [10]. More detailed descriptions are presented on the description sheet.

Material category	Column id*	Specific materials included
Aerated concrete (loose)	aconcrete_l	Aerated concrete in loose form, crushed.
Aerated concrete (solid)	aconcrete_s	Aerated concrete in solid blocks, slabs etc.
Aluminum	aluminium	Aluminum in extruded profiles etc. in windows and doors.
Bitumen felt	bitumen	Bitumen felt used as roof covering.
Brick	brick	Bricks of all kind, typically of clay, but may also include calcium silicate bricks.
Concrete	concrete	Concrete, including all types except aerated.
Expanded clay (loose)	expclay_l	Expanded clay in loose form, aggregate/pebbles.
Expanded clay (solid)	expclay_s	Expanded clay in solid blocks, slabs etc.
Glass	glass	Glass sheets in windows and some doors.
MDF	mdf	Medium-density fibreboard (MDF).
Mineral wool (hard)	minwool_h	Mineral wool in hard solid sheets.
Mineral wool (loose)	minwool_l	Mineral wool in loose, low density sheets or blown.
Mortar	mortar	Mortar in brick or block structures.
Plasterboard	plasterboad	Plasterboard.
Polystyrene insulation	polystyrene	Polystyrene insulation of all types.
Steel	steel	Steel including reinforcements, façade anchors, and major structural components such as beams.
Wood (solid)	wood_s	Wood, solid timber including planks, beams, battens, glued laminated timber (glulam).
Woodchip insulation	woodchip	Woodchips, sawdust or a mixture of them used as loose insulation.
Wood fiber insulation (solid)	woodfiber_s	Solid woodfibre sheets used as insulation.
Wood products	woodprod	Wood-based products such as plywood, chipboard, oriented strand board (OSB), excluding MDF and glulam.

⁎ In the dataset, column id appears at the end of a column header as described in Table 4.

**Table 2:** Combinations of construction decade, building type, bearing material and façade material included in the dataset [10].

Building type	Bearing material	Facade material	1940s	1950s	1960s	1970s	1980s	1990s	2000s	2010s
One dwelling house	Wood	Wood	D	D	D	T	D	D	D	D
One dwelling house	Wood	Brick		T	T	D	T	T	T	T
One dwelling house	Brick	Brick			D	D	D*	D	D	D
One dwelling house	Concrete	Concrete			D	T	D	D	D	D
One dwelling house	Concrete	Brick			T	D	T	T	T	T
Block of flats	Concrete	Concrete			D	D	D	D	D	D
Block of flats	Concrete	Brick			T	T	T	T	T	T

D = Direct record based on construction documents.

T = Theoretical variant with alternative façade material based on typical contemporary construction practice.

⁎ Geometry determined based on construction documents from 1979 due to lack of suitably sized cases dated in the 1980s, structures adjusted to match the represented decade.

**Table 3:** Distinctions between building parts included in the dataset [10].

Level of aggregation (sheet in dataset)	bp_level	bp_part	Notes on records
Entire building (data_building)	total	total	

Building level (data_building_level)	foundations	total	Piles below foundation bases not recorded due to site-specific variation and often insufficient information. Ground backfill not recorded. Frost insulation or waterproofing outside building perimeter not recorded.
	basement	total	Distinctions between basement and regular storey have been made based on annotations in buildings’ documents. Basements do not contain dwellings in blocks of flats or habitable rooms in one dwelling houses. In addition to being fully below ground, a basement can be located partially or fully above ground.
	storey[#]	total	Storey numbering for each building starts at 1, regardless of the numbering used in the original building documents, with 1 being the lowest non-basement storey.
	roof	total	Also includes any ceiling materials of the level below and any attics that are not included in the building's total floor area.

Building part (data_building_part)	foundations	total	As above.
	basement, storey[#]	floor	Also includes any ceiling surface materials of the level below. Vinyl floor covering, carpet, tiles, laminate etc. not recorded due to generally insufficient case specific information.
	basement, storey[#]	ext.walls	Windows and doors recorded separately (see below).
	basement, storey[#]	int.walls	Doors recorded separately, interior windows not found in sample. Tiles and grout not recorded due to generally insufficient case specific information.
	basement, storey[#]	balconies	Only found in blocks of flats.
	basement, storey[#]	chimneys	Firewalls that are not part of the chimney recorded under *int.walls*. Fireplaces not recorded due to generally insufficient information. All chimney materials, including those in the attic and above the roof, recorded as belonging to the building level the chimney starts from. Only found in one dwelling houses.
	basement, storey[#]	windows	Records based on typical reference windows for each decade and building type due to generally insufficient case specific information. Materials scaled from references based on window area (for e.g. glass) or perimeter (for e.g. frame).
	basement, storey[#]	ext.doors	Records formed as with windows.
	basement, storey[#]	int.doors	Records formed as with windows.
	roof	total	As above.

A total of 26 residential buildings’ direct material records are included (‘D’ in Table 2) which were derived from existing buildings. Here, bearing material refers to the main load-bearing material used in the vertical bearing members of the building above ground, and façade material refers to the main surface cladding of the exterior walls (ignoring possible tiles, plaster, or paint). The records represent the buildings in their originally designed state, without any possible later alterations. Additionally, based on these buildings, corresponding theoretical variants with the second most common façade material for the cohort (‘T’ in Table 2) were created by changing the surface material and any related parts, such as brick ties or battens. The 1940s one dwelling house has a solid log structure and thus no façade variant, as such buildings exists almost exclusively with wooden façades. All brick houses also had brick façades. As a result, in all 45 building cohorts are included.

**Fig. 1:**
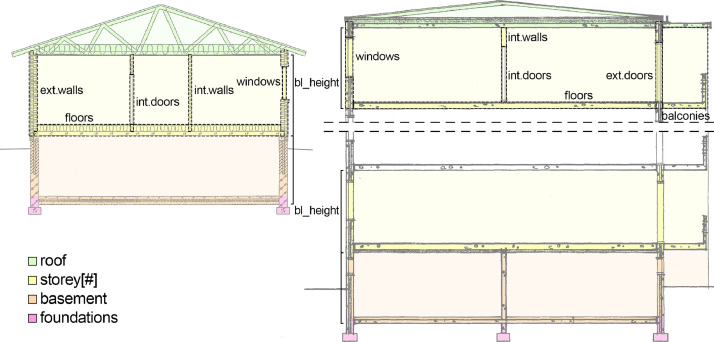
Building parts distinguished in the dataset [10]. For multi-storey buildings, records are included for all above-ground storeys individually. For building parts see Table 3, for bl_height Table 4.

In the dataset, each building (Table 2) is divided into three hierarchical degrees of aggregation: the entire building (*data_building*), building level (*data_building_level*), and building part (*data_building_part*). The entire building comprises all materials recorded for the case, building level distinguishes materials on each vertical level (foundations, basement, storey[#], roof) within a given building, and building part further distinguishes between e.g. floors and exterior walls within each level, where applicable (see Table 3 and Fig. 1).

### 3.2 Structure of the Sheets

Within the hierarchy described above, on each sheet the material records are further divided into *building structure* and *complementary building components. Building structure* includes all inventoried construction materials that are part of foundations, walls, floors, the roof, etc. but not windows or doors. Due to a lack of consistent information, the following categories are excluded: fasteners (aside from brick ties); membrane layers such as vapor barriers; and surface finish materials such as wallpaper, paint, and ceramic tiles. *Complementary building components* encompass materials found in doors and windows, including their frames. The distinction was made because the available documents only had consistently detailed information on the building structure, while only the nominal height and width were usually available for doors and windows. Correspondingly, the material records for the building structure are directly based on specific buildings’ construction drawings, while material records for doors and windows are based on their nominal dimensions and descriptions of typical components found in literature. The literature is detailed on the *description* sheet. To account for the impact of different geometries of components when applying typical material volumes, the figures for linear parts (e.g. frames, stiles) were scaled in relation to a component's nominal perimeter length, and planar parts (e.g. sheets of glass) in relation to its nominal surface area. Materials in other complementary building components such as ducts and pipes, wiring, building services equipment, gutters, and ladders are not included due to a general lack of sufficient information in the available documents.

Each row in the *data* sheets describes a single building, building level, or building part, as indicated by the names of the sheets. Additional information on them as well as the material records are presented in a range of columns as described in Table 4. In the application of the presented material intensities (MIs), it is very important to note the Finnish definitions of gross floor area (GFA) and total floor area (TFA) used in the dataset. Both are calculated along the outer surfaces of exterior walls, but GFA only includes areas that “have or could have spaces corresponding to the primary function of the building based on their location, connections, size, amount of natural light and other properties”[11] (authors' translation). Typically, this means that some storage spaces in basements or attics may be excluded from GFA. In some of the building cases used for this dataset, too, there are basements that, despite containing materials, do not contribute to the building's GFA. TFA, on the other hand, is what in international literature is commonly referred to as GFA, when a description is given (e.g. Heeren & Fishman [5]).

**Table 4:** Information included in the dataset [10] for each building part. Building parts included are further detailed in Table 3 and Fig. 1, materials included ([material]) in Table 1.

Column id	Content description
b_id	Identification code, used to group different building parts of a specific building.
b_type	Building type (residential block of flats, residential one dwelling house).
b_completionyear	The year when the construction of the building was originally finished. Recorded on a per-building basis even if e.g. foundations were finished in an earlier year than the roof.
b_datayear_oldest	The year from which the oldest documents used in the inventory are from.
b_datayear_newest	The year from which the newest documents used in the inventory are from. Documents from e.g. later renovations were only used to identify the original properties, not to include any later changes.
b_structuralsystem	Main vertical load-bearing structural system used in the building.
b_bearingmaterial	Main vertical load-bearing material of the building by mass. Recorded on a per-building basis even if e.g. the roof of a mainly concrete building is made out of wood and steel.
b_facadematerial	Main exterior surface material of the building's exterior walls by mass. Recorded on a per-building basis.
b_storeys	Number of storeys in the building. Possible attic or/and basement indicated by +a and/or +b respectively, and not included in the number of storeys.
b_gfa	Gross floor area of the entire building in accordance with the Finnish definition [12], measured along the exterior surfaces of exterior walls. Excludes spaces in basements and attics that are not “dwelling or working rooms or other space conforming to the principal intended use of the building”, i.e. some storage and technical spaces.
b_tfa	Total floor area of the entire building in accordance with the Finnish definition [13]. Otherwise the same as gross floor area but includes all spaces also in basements and attics. Corresponds to *external area* as defined by The Council of European Geodetic Surveyors [14], and common international definitions of gross floor area [5].
bl_height	Height of the building level, measured from the bottom of the floor to the bottom of the floor or ceiling above (see Figure 1 for illustration). For levels with varying heights, the value is an average weighted based on corresponding floor areas. Does not include, and not recorded for, foundations or roofs.
bl_gfa	Gross floor area of the building level. For foundations, this equals the area of the bottommost storey(s) under which there are foundations. For roofs, this equals the area of the topmost storey(s) over which there is a roof.
bl_tfa	Total floor area of the building level. Defined as described above for b_gfa, b_tfa and bl_tfa regarding the the respectively relevant parts.
bp_level	Vertical level of the building part, described on the current row. See Fig. 1 for illustration.
bp_part	The building part described on the current row. See Table 3 for description and Fig. 1 for illustration.
mi_gfa	Total material intensity for the row's extent of records calculated in relation to gross floor area, kg/m^2^_GFA_.
mi_tfa	Total material intensity for the row's extent of records calculated in relation to total floor area, kg/m^2^_TFA_.
mi_s_gfa_[material]	Material specific material intensity for the row's extent of records in the building structure, kg/m^2^_GFA_.
mi_c_gfa_[material]	Material specific material intensity for the row's extent of records in complementary building components, kg/m^2^_GFA_.
mi_s_tfa_[material]	Material specific material intensity for the row's extent of records in the building structure, kg/m^2^_TFA_.
mi_c_tfa_[material]	Material specific material intensity for the row's extent of records in complementary building components, kg/m^2^_TFA_.
m_s_[material]	Total mass of a material for the row's extent of records in the building structure, kg.
m_c_[material]	Total mass of a material for the row's extent of records in complementary building components, kg.
v_s_[material]	Total volume of a material for the row's extent of records in the building structure, m^3^.
v_c_[material]	Total volume of a material for the row's extent of records in complementary building components, m^3^.

The *description* sheet contains the following: a detailed description of the types of information in the *data* sheets, as also summarised in Table 4; building parts distinguished (Table 3); materials included (Table 1); building parts, components, or products excluded from the records; material densities used to calculate masses from volumes along with the corresponding references; component types and references used to calculate material volumes in windows and doors.

## 4 Experimental Design, Materials and Methods

### 4.1 Representative Building Selection

The buildings underlying this dataset were sampled from Vantaa, the fourth largest city in Finland. This is because thanks to a collaborative research project, the researchers had access to extracts of the City of Vantaa's building register and digital archives of the local building inspection authority, which would have otherwise carried substantial fees. The buildings contained in the current dataset were sampled from Vantaa's building stock in 2018 (N = 27,695 buildings) using a representative sampling approach that considered the following properties: residential building type, construction year, main bearing material, main façade material, prevalence in the stock, gross and total floor area, and number of storeys.

To keep the workload manageable, rarer residential building types, such as attached two-family houses (15% of the stock by number, 10% by TFA) and row houses (11% of the stock by number, 13% by TFA), were excluded, and only one dwelling houses and blocks of flats were covered. Construction year was considered to account for building codes, and construction practices in general, changing over time. Although these changes happen at varying intervals, a decade based categorization was chosen for its simplicity and intercompatibility with existing statistics databases such as those of Statistics Finland [15]. Main bearing and façade materials were considered as they greatly affect the amounts and shares of materials found in a building. Of these the bearing material is more permanent than the façade material, thus more likely to still hold true for the oldest cohorts, and therefore the primary criterion. Prevalence of buildings in the cohort was used to focus resources on the most common types found. On average, a building with a larger floor area has a lower material intensity than a smaller one, especially if its exterior walls are bearing [4]. Similarly, for a given amount of floor area, more storeys corresponds to a smaller footprint and thus less materials in the bottom floor, foundations, and roof. Therefore, in case selection values at or close to the median were sought in order to minimize building geometry based error when applying the data to buildings of different sizes in the stock.

First, the building cohorts to be represented in the material inventory dataset were identified from the entire Vantaa building stock as follows (in parentheses: number of cohorts included after each step):

• building type: one dwelling houses and blocks of flats (2)
• construction time: by decade, 1900–2019 (24)
• main bearing material: wood, brick, concrete (72)
• prevalence: cohorts with under 20 buildings excluded (32)

The prevalence criterion led to the exclusion of (based on bearing material) brick one dwelling houses from before the 1960s, concrete one dwelling houses from before the 1950s, and all non-concrete blocks of flats.

Second, for each of the 32 cohorts identified, a small number of representative case buildings were identified from the Vantaa building register considering the cohort medians for TFA and number of storeys in the buildings.

Third, drawings and documents were sought for these case buildings from the building inspection authority's digital archives [16]. Due to a lack of documents in the archives, all cohorts built before the 1940s had to be excluded (3.8% of Vantaa stock for one dwelling houses, 0.1% for blocks of flats), as well as concrete cohorts from the 1950s (1.2% of Vantaa stock for one dwelling houses, 0.9% for blocks of flats). This led to the final sample described in Table 2. Finally, for each building type–construction decade–main bearing material cohort identified, one representative case building with (1) the most common façade material, and (2) a sufficient quality of drawings and documentation was selected for direct material inventorying, the process of which is described in the next sections.

However, the following exceptions to the general rules should be noted. First, instead of specific case buildings, the materials of one dwelling houses from the 1940s and 1950s were recorded based on type-planned houses, the drawings of which were acquired from the National Archives of Finland [17]. This was because drawings for such buildings were not available in the Vantaa archives. However, the type-planned building designs were widely used in Finland for the post-WWII reconstruction up until the end of the 1950s. Second, because drawings were not available for a median-sized house from the 1980s, the building geometry of the 1980s brick one dwelling house was determined using a house from 1979 and adjusting insulation thicknesses based on construction drawings of actual 1980s houses to match the building code at force in the 1980s. Third, in some of the one dwelling houses categorised as concrete, the bearing structure and/or façade were in fact made of aerated concrete or expanded clay blocks. However, the categorisation as concrete is also used in the city's official building register and was thus retained for the purposes of the current dataset. In the material data of the current dataset, the materials are distinguished as described in Table 1.

### 4.2 Source Material Acquisition and Types

Already digitised construction drawings and specification sheets were used in the PDF format. They were downloaded primarily from Vantaa building inspection authority's digital archives through the online purchase portal Lupapiste Kauppa [16]. For wooden one dwelling houses from the 1940s and 1950s, drawings and other documentation (such as original bills of materials) of type-planned houses were downloaded from the Open Access digital archives of the National Archives of Finland [17]. The specific documents available varied from building to building, typically comprising floor plans, elevations, and sections in scale 1:100, complemented by detail drawings in scale 1:5–1:20 on e.g. structural layers and their joints, foundations, and trusses. All documents in the building inspection authority's archive include a permanent building identification number. This number was used to sort the acquired documents and ensure that the documents used for the material inventory were for the correct building even when this was not visually apparent, as was the case for e.g. some structure type descriptions. In addition to the aforementioned documents, literature on typical design solutions as well as various design standards and guidance were reviewed to account for any missing information in some buildings’ drawings, considering e.g. wall stud spacing and dimensions. Drawings of structurally similar buildings from matching cohorts were also used for the same purpose.

### 4.3 Measurement Protocol, Records Format and Recording Process

Material types and volumes were primarily determined based on the construction drawings obtained. When needed to account for lacking annotations on dimensions, scale drawings were imported into a computer-aided design (CAD) programme (Graphisoft ArchiCAD or Autodesk Revit) and measured. The specific methods of measurement varied including simple linear measurements, area-based measurements, and volume-based modelling depending on which was deemed most practical and accurate for the building part and material in question. For some of the wood framing, the AGACAD plugin for Revit, an aid for 3D modelling timber structures, was used.

During this process, the construction drawings were cross-referenced with one another and as needed with other documents, such as bills of materials where available, to determine the volume of each inventoried material (in m^3^). The material volumes were then recorded in the datasheets in Microsoft Excel. When original bills of materials were used, the stated excess amount (typically 10%) to account for offcuts, and other material losses during construction, was excluded. During and after the recording process, the documents and resulting records (directly in numerical form and via charts) were reviewed by the authors together and independently to avoid misinterpreting the documents and identify input errors.

For the theoretical brick façade variants, the existing wooden or concrete façade cladding layer and any related wooden battens or steel trusses were replaced with a layer of perforated 85 mm façade bricks, mortar, and brick ties. All other layers, including the original bearing part of the wall and insulation, were kept the same, as would be the case based on existing corresponding mixed material walls. In the two cohorts where brick was the most common cladding material (1970s wood-framed and concrete one dwelling houses), an equivalent process was used to create the theoretical wood and concrete façade variants.

Unless otherwise indicated in the documents available, all brick walls were assumed to use standard perforated clay bricks: 285 mm long, 60 mm tall and 85 mm (façade claddings) or 130 mm (all others) wide depending on wall dimensions. Correspondingly, by default mortar seams in such walls were assumed to be 15 mm thick. In the material records of the dataset, the holes in perforated bricks have been subtracted so that the material volumes represent the actual amount of fired clay. For reinforcement steel, any extra length needed for lap splices has not been included when calculating running meters and corresponding material volumes.

After recording the material volumes, building part-specific masses for materials were calculated using Eq. 1:

(1) $$\begin{array}{c} M_{m,\mathrm{bp}}=V_{m,\mathrm{bp}}\times \rho_{m} \end{array}$$

where *M_m,bp_* is the mass of material *m* in building part *bp* (in kg), *V_m,bp_* is the volume of material m in building part *bp* (in m^3^), and *ρ_m_* is the density of material *m* (in kg/m^3^). Typical material densities were obtained mainly from the Finnish national LCA database CO2data.fi [18].

Building part- and material-specific material intensities were calculated using Eq. 2:

(2) $$MI_{m,bp}=\frac{M_{m,bp}}{A_{bl}}$$

where *MI_m,bp_* is the material intensity of material *m* in building part *bp* (in kg/m^2^ of gross or total floor area), *M_m,bp_* is the mass of material *m* in building part *bp* (in kg), and *A_bl_* is the (gross or total) floor area of the building level the building part belongs to (in m^2^).

Aggregate figures for all materials and/or at the scales of specific building levels or buildings were calculated using the same principles, so that e.g. the material intensity of an entire building level is the sum of all material masses on that level divided by the floor area (TFA or GFA) of the level.

## Ethics Statements

The data or data collection does not involve human subjects, animal experiments or social media platforms.

## CRediT authorship contribution statement

**Tapio Kaasalainen:** Conceptualization, Methodology, Investigation, Data curation, Validation, Writing – original draft, Writing – review & editing. **Mario Kolkwitz:** Conceptualization, Methodology, Investigation, Data curation, Validation, Writing – review & editing. **Bahareh Nasiri:** Methodology, Investigation, Resources, Writing – review & editing. **Satu Huuhka:** Conceptualization, Methodology, Investigation, Writing – review & editing, Project administration. **Mark Hughes:** Writing – review & editing, Funding acquisition.

## Data Availability

Building part specific material inventory dataset for residential buildings in Finland (Original data) (Zenodo).
